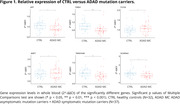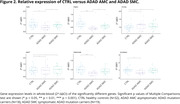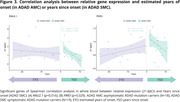# Analyzing the expression of candidate genes in whole blood of autosomal dominant Alzheimer’s Disease

**DOI:** 10.1002/alz.089842

**Published:** 2025-01-03

**Authors:** Aina Comas‐Albertí, Oscar Ramos‐Campoy, Agnès Pérez‐Millan, Bea Bosch, Alba Gómez‐Núñez, Roger Puey, Guadalupe Fernandez‐Villullas, Mircea Balasa, Albert Lladó, Anna Antonell, Raquel Sánchez‐Valle

**Affiliations:** ^1^ Alzheimer’s disease and other cognitive disorders Unit. Hospital Clínic de Barcelona; FRCB‐IDIBAPS; University of Barcelona, Barcelona Spain; ^2^ Hospital Clínic de Barcelona ‐ Fundació de Recerca Clínic Barcelona – IDIBAPS ‐ University of Barcelona, Barcelona, Catalonia Spain

## Abstract

**Background:**

Autosomal dominant Alzheimer’s Disease (ADAD) represents around 0.5% of all AD cases, and is caused by mutations in *PSEN1*, *PSEN2* and *APP* genes. Gene expression studies can be useful for unravelling the physiopathology of AD and identifying potential biomarkers. However, most studies are focused on late‐onset AD (LOAD), and mainly on brain tissue or immune cells. Preliminary data from our group showed 9 common differentially expressed genes (DEGs) both in brain tissue and lymphoblastoid cell lines in ADAD versus healthy controls (*AMMERCR1L, FMR1, FXYD5, PRR3, RNU2.1, SCARNA2, SLC35A1, TCEAL8, TMEM184B*). We aim to investigate these DEGs along with 3 AD relevant genes (*APP, PSEN1* and *MAPT*) in whole blood of ADAD with the goal to explore new potential biomarkers.

**Method:**

Our cohort (N = 69) consisted of ADAD symptomatic mutation carriers (SMC; N = 19), asymptomatic mutation carriers (AMC; N = 18) and healthy non carriers (CTRL; N = 32). RNA was extracted from whole blood and relative gene expression was determined by RT‐qPCR and ΔΔCt method. Permutation tests were used to determine the differential gene expression between groups. Additionally, a correlation analysis was performed between estimated years of onset (for AMC) or years since onset (for SMC) and relative expression.

**Result:**

DEGs were mainly down‐regulated in ADAD with respect to CTRL, except for *FXYD5* and *MAPT*, which were up‐regulated. When comparing ADAD mutation carriers versus CTRL the significant genes were *APP* (p = 0.045), *FXYD5* (p = 0.018), *MAPT* (p = 0.041), *PSEN1* (p = 5e‐4), *SCARNA2* (p = 0.042) and *TMEM184B* (p = 0.0042) (Fig. 1). Then, we compared AMC versus CTRL and SMC versus CTRL and found two DEGs in both comparisons: *FXYD5* (p = 0.015; p = 0.033) and *MAPT* (p = 0.015; p = 0.034). In AMC group we also found these DEGs: *FMR1* (p = 0.011), *PRR3* (0.029), *PSEN1* (p = 2e‐4), *SCARNA2* (p = 0.027) and *TMEM184B* (p = 5e‐4) (Fig. 2). Results showed a significant negative correlation for *RNU2.1* (p = 0.016) and *PRR3* (p = 0.029) between relative expression and years since onset in SMC (Fig. 3).

**Conclusion:**

Mutation carriers show some DEGs in whole blood, even in the asymptomatic stage. *FXYD5* and *MAPT* genes show a consistent up‐regulation in both SMC and AMC, suggesting it could be further validated as a possible genetic biomarker in whole blood of ADAD.